# Attachment and Depressive Symptoms in Adolescence: The Mediatory Role of Emotion Awareness

**DOI:** 10.3390/bs12100405

**Published:** 2022-10-21

**Authors:** Maria João Carapeto, Raquel Domingos, Guida Veiga

**Affiliations:** 1 Departamento de Psicologia, Escola de Ciências Sociais, Universidade de Évora, 7005-345 Évora, Portugal; 2 Departamento de Desporto e Saúde, Escola de Saúde e Desenvolvimento Humano, Universidade de Évora, 7005-345 Évora, Portugal; 3 Comprehensive Health Research Centre (CHRC), Universidade de Évora, 7005-345 Évora, Portugal

**Keywords:** internalizing, differentiation of emotions, bodily awareness, emotion regulation

## Abstract

Attachment seems to influence depression through emotion regulation. However, no study has yet examined the mediatory role of emotion awareness, a particular subset of emotion regulation abilities, in the relationship between attachment and depressive problems in early and middle adolescence. The aim of this study is to examine the direct and indirect effects of attachment on depressive symptoms in adolescence, considering the mediatory role of emotion awareness dimensions. A sample of adolescents (*n* = 223) filled up self-report questionnaires on attachment, emotion awareness and depression. Serial mediation models suggest direct effects on depression: negative for secure attachment and positive for anxious/ambivalent attachment. Anxious/ambivalent attachment has a positive indirect effect through lower differentiation of emotions. Both secure and anxious/ambivalent attachment have indirect positive effects on depression through the sequence of bodily unawareness and differentiation of emotions. Differentiating emotions has a central role in mediating the relationship between attachment and depressive symptoms, and the lack of bodily awareness of emotions contributes to such mediation.

## 1. Introduction

In current societies, depressive symptoms seriously threaten adolescents’ mental health and socioemotional adjustment [1]. In this context, research about a diversity of risk factors and mechanisms for depression has been encouraged [1,2,3,4]. Therefore, this article focuses on the possible contribution of attachment and emotion awareness to depression in early to late adolescents.

The attachment theory [5,6] highlights the importance of a child’s early relationships with caregivers for the child’s survival, adaptive development, and mental health. Based on his/her experience with the caregiver, the child construes internal working models, which are a mental representation of the self and significant others that perdure and inspire personal behavior, cognition, affect, and emotion regulation [5]. Three main patterns of attachment that differently guide individuals’ actions in times of distress, have been described [5,6,7,8]. Secure attachment representations promote a sense of security and trust in relationships and a sense of being worthy of others’ attention [5,7]. Insecure anxious/ambivalent attachment representations are related to a continuous search for proximity and closeness, concerns about relationships, and fear of reject [7,8]. Insecure avoidant attachment representations focus on self-reliance and a preference for emotional distance from others [7,8].

Insecure attachment (more consistently, the anxious/ambivalent attachment) has been related to depressive symptoms [6,9,10] in both clinical and non-clinical adolescents [10]. On the other hand, secure attachment has been related to better adjustment, including fewer depressive problems [10]. This relationship between attachment and depressive symptoms seems to be mediated, at least partially, by emotion regulation [11,12], which refers to the “extrinsic and intrinsic processes responsible for monitoring, evaluating, and modifying emotional reactions, especially their intensive and temporal features, to accomplish one’s goals” [13] (pp. 27–28). Emotion regulation processes are first shaped by early attachment relationships, in which the caregiver supports the child to become increasingly attuned with his/her own emotions and behaviors, decreasing the emotional arousal and improving his/her sense of comfort and safety [5,7,8]. Accordingly, insecure attachments seem to favor maladaptive emotion regulation strategies in response to distress (e.g., hyperactivating and deactivating strategies) [8,12,14]. In contrast, secure attachment is related to fewer maladaptive [8] and more adaptive emotion regulation strategies [15]. Moreover, emotion regulation difficulties are linked to adolescents’ depression [16,17].

Most studies about the mediation role of emotion regulation in the relationship between attachment and depressive symptoms in adolescence [11,12] have approached emotional regulation as a process of “modifying emotional reactions” [13]. Therefore, they have neglected the “monitoring and evaluating” component, the so-called emotion awareness. Emotion awareness is an attentional process, involving interpretative and evaluative functions, that “not only enables us to monitor our emotions but also to differentiate between various emotions in a qualitative sense; to locate their antecedents; and to acknowledge the physiological correlates of the emotional experience for what they are” [18] (p. 756). Emotion awareness includes attitudinal aspects, namely the positive or negative value one attributes to emotions, the understanding of emotions as experiences more or less private to share (or to hide) and as a more or less interpersonal experience (communication of personal emotions and attention to others) [18]. Emotion awareness is, therefore, a multidimensional construct.

Emotion awareness seems to play a critical role in what concerns the development of adolescents’ depressive symptoms [16,19,20,21]. That is, the diminished ability to identify which emotion one is feeling or what causes it hampers the ability to modify emotional reactions, leading to persistent negative emotions and increasing the likability of developing depressive problems [19,22,23]. On the other hand, some research showed that adolescents’ secure attachment is related to better emotion awareness [15] and that insecure attachment is linked to difficulties in emotion awareness [8,24,25].

To date, only three studies have been conducted on the subject, and these showed that emotion awareness has a mediatory role in the positive relationship between insecure (more frequently avoidant) attachment and depression in college students [24] and adult women [26,27].

### Present Study

Despite the empirical support for the relationships between attachment, emotion awareness, and depressive symptoms in adolescents, to the best of our knowledge, this is the first study to examine the mediatory role of emotion awareness in the relationship between attachment and depressive problems in early and middle adolescence. Moreover, several developmental changes occur at this stage, making it critical to understand the contribution of attachment and emotion awareness to adolescents’ mental health, particularly concerning depressive symptoms: (1) The attachment system becomes more committed to emotion regulation functions as adolescents face autonomy challenges and new social contexts [28]; (2) Emotion regulation abilities seem to deteriorate [29], including emotion awareness [19,25]; (3) Depressive symptoms increase [1]. Moreover, this is also the first study to consider emotion awareness as a multidimensional construct, when examining its mediatory role in the relationship between attachment and depressive symptoms.

Thus, the present study aims to examine the direct and indirect effects of attachment patterns (secure, anxious/ambivalent, and avoidant) on depressive symptoms in early and middle adolescents, considering the mediatory role of different emotion awareness dimensions. As age and gender seem to have an influence on depression [1] and emotion awareness [19], and considering the recognized overlap and comorbidity between anxiety and depression [30], the possible effects of gender, age and anxiety will be controlled. Based on previous studies with older ages, it is expected that emotion awareness plays a mediatory role in the positive relationship between insecure attachment and depression, as well as in the negative relationship between secure attachment and depression. Giving that this is the first study to consider the multidimensionality of emotion awareness, no hypotheses are formulated regarding the role of each emotion awareness dimension.

As main contributions, we expect this work to clarify some of the mechanisms that lead to adolescents’ depressive symptoms, particularly those involving attachment and emotion awareness. Moreover, we expect these findings will provide new insight to improve the prevention and treatment of adolescents’ depression.

## 2. Materials and Methods

### 2.1. Participants

The sample consists of 223 participants, 96 (43%) male and 127 (57%) female, aged between 12 and 16 years old (M = 13.51, SD = 1.13), who attended the 7th grade (*n* = 83, 37.2%), 8th grade (*n* = 69, 30.9%), and 9th grade (*n* = 71, 31.8%) in 3 public schools in the South of Portugal. Most participants reported never having lived apart from their parent(s) (74.9%) and currently living with both their parents (76.7%) or only with their mother (20.6%). The majority reported having a single brother or sister (63.7%) and 14.8% had no brothers or sisters. Concerning educational qualifications, 26.9% reported that fathers had secondary education and 35.9% higher education; 30.5% indicated that mothers had secondary education and 46.6% higher education.

### 2.2. Instruments

The Childhood and Adolescence Attachment Inventory (CAAI) [31] is a self-report questionnaire that measures three attachment patterns. The secure attachment scale measures issues of trust in the other and one’s abilities (e.g., I like feeling close to other people). The anxious/ambivalent scale measures apprehension and concerns about relationships (e.g., I am concerned about the possibility of being alone), while the avoidant attachment scale measures dependence and avoidance (e.g., I am concerned if I have to depend on other people) [31]. Each scale comprises 8 items to be answered on a 5-point Likert scale, from 1 (*Never*) to 5 (*Always*), and its score is obtained through the sum of each item scores. Higher scores reflect a higher level of attachment behaviors and representations on an attachment scale. The CAAI showed good psychometric qualities, such as adequate adjustment measures of the model in confirmatory factor analysis and good reliability (α = 0.83; 0.85; 0.71 for secure, insecure-anxious/ambivalent, and insecure-avoidant scales, respectively) [31]. In the present study, the reliability of the secure, insecure-anxious/ambivalent, and insecure-avoidant scales was good (α = 0.73; α = 0.84; α = 0.63, respectively).

Emotion awareness was measured using the Portuguese version [32] of the Emotion Awareness Questionnaire [18]. This self-report questionnaire comprises 30 items corresponding to six scales: differentiation of emotions (7 items; e.g., “I am often confused or puzzled about what I am feeling”), verbal sharing of emotions (3 items; e.g., “I find it difficult to explain to a friend how I feel”), not hiding emotions (5 items; e.g., “Other people don’t need to know how I am feeling”), bodily unawareness of emotions (or lack of bodily awareness) (5 items; e.g., “When I am scared or nervous, I feel something in my tummy”), attending to others emotions (5 items; e.g., “It is important to know how my friends are feeling”), and analysis of own emotions (5 items; e.g., “When I am angry or upset, I try to understand why”). Items are scored on a 3-point Likert scale from 1 (*not true*) to 3 (*often true*), and the scoring is reversed for 20 items. Each scale score is the average score of its items and higher scores correspond to better emotion awareness and are associated with less negative affective states [18]. The Portuguese version showed good psychometric qualities [32]. In the present study, the reliability of the scales was good (0.66 < α < 0.74).

The Depression Anxiety Stress Scale—21 [33,34] was used to measure depression and anxiety. In this self-report questionnaire, items are scored on a 3-point Likert scale from 0 (*did not apply to me at all*) to 3 (*applied to me very much, or most of the time*). The depression scale score is obtained through the sum of the scores of its 7 items (e.g., I couldn’t seem to experience any positive feeling at all) multiplied by 2, and the anxiety score is the sum of the scores of its 7 items (e.g., “I was aware of dryness of my mouth”) multiplied by 2. Higher results are associated with higher severity of symptoms. The Portuguese version showed good psychometric qualities [34]. In the present study, the reliability of the depression and anxiety scales was good (α = 0.85 and α = 0.81, respectively).

### 2.3. Procedure

This research project was approved by the Ethics Committee of the University of Évora (#18003) and the Portuguese Ministry of Education. Before conducting the study, permission to collect the data was obtained from schools’ principals, and adolescents and parents provided written informed consent.

### 2.4. Data Analysis Strategy

The statistical analysis was carried out with the SPSS v. 24. Descriptive statistics (mean and SD) were computed for age, attachment patterns (secure, anxious/ambivalent and avoidant), emotion awareness dimensions (differentiation emotions, verbal sharing of emotions, not hiding emotions, bodily unawareness of emotions, attending to other’s emotions, and analysis of own emotions), depression and anxiety, as well as gender (%). A series of *t*-tests for independent samples were computed to test gender differences and Pearson correlations were calculated between age, attachment patterns, emotion awareness dimensions, and depressive and anxiety symptoms.

Multiple mediation models were considered to test the hypotheses that attachment patterns influence depressive symptoms and that this influence is mediated by emotion awareness. Two emotion awareness dimensions, namely differentiating emotions and bodily unawareness presented the highest, medium size (>0.30) correlations with depression and were selected as mediation variables. Considering the medium size association between the two mediators (*r* = 0.35), a serial multiple mediation model was the option [35].

**Figure 1:**
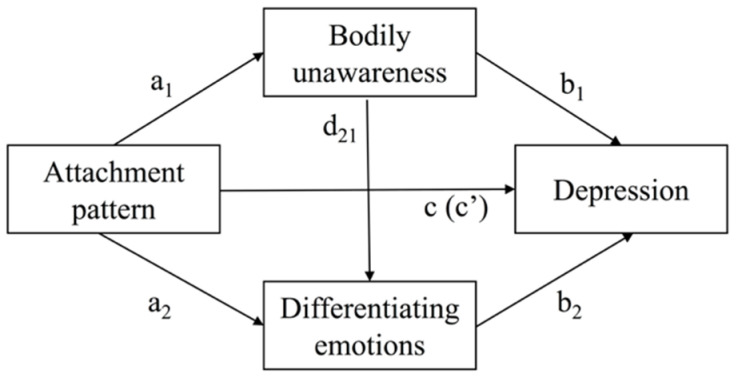
General serial multiple mediation model.

Analyses were performed with Hayes’ Process v. 3.5 macro for SPSS [35], a software that uses a path analyses approach and is based on the regression ordinary least squares method. Based on model 6 (Figure 1) and following Hayes [35] guidelines, the serial multiple mediation models included eight predictors (three attachment patterns; two mediators, namely bodily unawareness and differentiating emotions; and three additional covariates, that is, gender, age, and anxiety) and depression as the outcome variable.

The analysis provided regression coefficients for each path in the models (e.g., a_1_, a_2_, b_1_, b_2_, d_21_) including the estimates of the total (c path; mediators not included in the model) and direct (c’ path; mediators included in the model) effects of each attachment pattern on depressive symptoms and the three possible indirect effects (a_1_b_1_, a_2_b_2_, and a_1_d_21_ b_2_ paths) for each attachment pattern (Figure 1). To compute the direct and indirect effects of each attachment pattern, the model run three times, each one considering one specific attachment pattern as the independent variable (the other two entering as covariates). To test the significance of the coefficients of each path (including indirect paths), 95% percentile bootstrap confidence intervals and bootstrap standard errors were computed based on 10,000 bootstrap samples. In addition, a heteroscedasticity-consistent method (i.e., HC3) was used to compute standard errors to reinforce more robust inference. An effect was considered significant when the confidence interval did not include zero. Otherwise, a result was considered statistically significant when *p* < 0.05.

## 3. Results

Table 1 shows the mean and standard deviation values for the variables and the correlations, and Table 2 presents the descriptives by gender.

The analysis of the correlations in Table 1 suggests that secure attachment was positively related to emotion awareness dimensions, except for differentiation of emotions and bodily unawareness. Both insecure patterns were negatively associated with differentiation of emotions and verbal sharing. However, only anxious/ambivalent attachment was negatively associated with body unawareness, and avoidant attachment was negatively related to not hiding of emotions.

Depression was negatively associated with secure attachment and positively associated with both insecure attachment patterns. Moreover, depression was negatively related to emotion awareness dimensions, except for analyzing emotions. Positive associations were found between anxious/ambivalent and avoidant attachment and depression and anxiety.

As shown in Table 2, females reported higher anxious/ambivalent attachment. Males showed higher scores on differentiating emotions and bodily unawareness, whereas females scored higher on attending to others’ emotions. Age was not associated with any variable.

**Figure 2:**
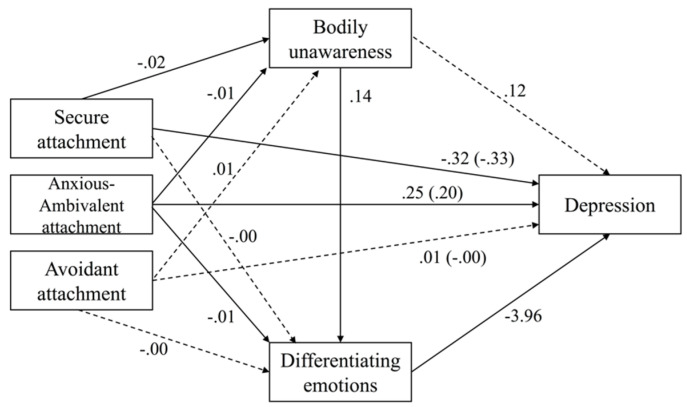
Serial multiple mediation models of the effects of bodily unawareness and differentiating emotions on the relationship between different patterns of attachment and depression. Unstandardized coefficients (direct effects in parentheses). Solid lines indicate significant effects (95% confidence interval does not include zero) and dashed lines illustrate non-significant effects.

The total, direct and indirect effects are presented in Table 3, and the coefficients for the remaining paths. Figure 2 illustrates the relationships between the variables.

The model of the total effects of attachment (age, gender, and anxiety were covariates) on depression explained 57% of the variance of depression, *F* (6, 216) = 45.92, *p* = 0.0000, and the model for the direct effects (mediators included) explained 60%, *F* (8, 214) = 38.54, *p* = 0.000.

The negative total (c path) and direct (c’ path) effects of secure attachment on depression indicated that more secure attachment predicted lower levels of depressive symptoms even when the emotion awareness dimensions entered the model. Besides, a positive indirect effect of secure attachment on depression via bodily unawareness and differentiating emotions (a_1_d_21_b_2_ path) was significant.

Anxious/ambivalent attachment showed positive total (c path) and direct (c’ path) effects on depression, such that more anxious/ambivalent attachment predicted higher levels of depression even when the effect of the mediators was considered. The total indirect effect of anxious/ambivalent attachment on depression was also positive, as well as two specific indirect effects, namely, through differentiating emotions (a_2_b_2_ path) and through bodily unawareness and differentiating emotions (a_1_d_21_b_2_ path). The first indirect effect was more expressive than the second, *b* = 0.039, *boot se* = 0.021, 95% *boot CI* [0.002, 0.084]. Avoidant attachment was not significantly related to emotion awareness dimensions nor depression.

Thus, secure and anxious/ambivalent attachments were negative predictors of bodily unawareness, and both anxious/ambivalent attachment and bodily unawareness were predictors (negative and positive, respectively) of differentiating emotions. The models with the attachment patterns as predictors (also gender, age, and anxiety as covariates) explained 23% of the variance of bodily unawareness, *F*(6, 216) = 11.12, *p* = 0.000, and (adding bodily unawareness to the model) 26% of differentiating emotions, *F*(7, 215) = 10.58, *p* = 0.000.

## 4. Discussion

The findings of the present study show that both secure and anxious/ambivalent attachment are related to depression and that bodily unawareness and differentiating emotions mediate those relationships.

In line with other studies [10], the significant direct effects in our study suggest that more secure attachment representations are linked to lower levels of depression, and more anxious/ambivalent attachment representations are related to higher levels of depression. Therefore, the influence of both secure and anxious/ambivalent attachment on depression seems to go beyond the mediated effects of the two emotion awareness dimensions (and beyond the effects of the covariates: gender, age, anxiety, and the other attachment patterns) that we discuss later. This is consistent with the proposed pervasive influence of attachment representations on the psychological organization [5,36] and with the diversity of mechanisms that have been proposed to mediate the influence of attachment on depression, such as dysfunctional attitudes, self-criticism, self-compassion, or maladaptive emotion regulation [11].

Emotion awareness seems to play a role in the pathway between attachment and adolescents’ depressive symptoms. In particular, as suggested by the serial mediation analyses, more secure and anxious/ambivalent attachments both predict greater levels of depression through differentiating emotions and bodily unawareness.

First, differentiating emotions emerges as the most relevant indirect pathway from anxious/ambivalent attachment to depression. The relationship between the insecurity of attachment and differentiating emotions has been found in other studies with adolescents [25] as well as the link between differentiating emotions and depression [18,21]. Possibly, the chronic concern of being disliked and rejected by others limits the opportunities to perceive a diversity of emotional states and to understand the different causes of emotions. This hampers the possibility of coping adequately with the specificities of the emotion-evoking situations; therefore, the initial negative affect may develop into depressive symptoms [22,23]. This is especially critical because adolescents are repeatedly distressed by daily developmental challenges and interpersonal stressors [21]. On the other hand, there is some evidence that the lack of differentiation of emotions and their causes predicts maladaptive forms of emotion regulation, such as brooding rumination [37], a recognized risk factor for depression [1], especially when emotion differentiation is low [38]. Brooding rumination is also a mediator between insecure attachment and depression [11].

A second indirect pathway from anxious/ambivalent attachment to depression involves, sequentially, bodily unawareness and differentiating emotions. Accordingly, more anxious/ambivalent attachment representations might increment the focus on the physiological arousal during emotional experiences [39], which might hinder an external focus and a refined differentiation of emotional experiences based on the clues of the emotion-evoking situations [18]. Altogether, this opens the pathways to depression as mentioned above. In addition, the hypervigilant focus on body sensations might limit identifying negative emotions-evoking situations. Hence, dysfunctional attitudes and self-criticism might come into play, therefore contributing to depressive symptomatology [11,40].

Third, although the direct effect supports the hypothesis of the secure attachment’s protective effect against depression [6,10], the indirect, serial path through both emotion awareness dimensions suggests a novel hypothesis that more secure attachment can also open a maladaptive pathway to depressive symptoms. Altogether, these findings suggest that trusting on own abilities and others (the core of secure attachment), and concerning about being rejected and disliked by others (the core of anxious/ambivalent attachment) are related to a higher focus on the physiological arousal throughout emotional processing and to a lower focus on the situations that evoke the emotional response. In this scenario, the entry into the scene of adaptive emotion regulation to cope with the situation seems less likely. In contrast, the maladaptive strategies (e.g., brooding rumination) seem more likely [37]. It is important to note that Rieffe and colleagues [18] had already adverted to the risk of high bodily awareness of emotions and the importance of a combined understanding of bodily awareness with other emotion regulation abilities in the process of emotional regulation. Such contradictory effects of secure attachment on depression support the idea of the complex, organismic nature of the socioemotional processes [36].

Finally, in line with other research [41], we find that avoidant attachment has no significant role in depressive symptoms. Hence, our findings do not support the relationship between avoidant attachment and greater depressive symptoms found by others [9,10,26,42], nor the hypothesis of the protective effect of avoidant attachment on depression [24] (see also [42]). Additionally, contrary to other studies [24,27], our findings do not support the influence of avoidant attachment on emotion awareness nor its indirect effect on depression through a lack of emotion awareness.

As mentioned by other authors (e.g., [42]), avoidant attachment’s role on depressive symptoms needs yet to be clarified. For instance, Khan and colleagues (2020) claim the need for a finer-grain analysis of the effects of two main types of avoidant attachment (i.e., dismissive and fearful). They hypothesize that the avoidant fearful type might be a fertile ground to adolescents’ depression, while the dismissive type might protect adolescents from depressive problems. Therefore, future studies should consider these two subgroups in order to disentangle the pathway from avoidant attachment to depression.

## 5. Conclusion

### 5.1. Limitations and Future Directions

Although encouraging, our findings need to be taken with caution. It is important to note that the cross-sectional design limits assertions about causality. Besides, testing the present models in other cultures would be worthwhile. Additionally, more studies are still needed to clarify the role of avoidant attachment. Our study reveals that bodily awareness of emotions, favored by both secure and anxious/ambivalent attachment representations, weakens the differentiation of emotions, thereby indirectly promoting adolescents’ depressive symptoms. However, more research is needed to unfold this process. Besides, longitudinal research is needed to further examine the role of emotion awareness dimensions, their interplay, and the intertwined with other emotion regulation strategies and cognitive-emotional processes.

### 5.2. Theoretical and Practical Implications

The present findings suggest that the pathways from adolescents’ attachment representations to depression are multiple and that different emotion awareness abilities are involved differently. Concerning implications for practice, secure and anxious/ambivalent attachment representations and the ability to differentiate emotions and their causes are important target variables for interventions to prevent and treat adolescent depression [43].

### 5.3. General Conclusions

Secure and anxious/ambivalent attachment representations are related to adolescents’ depressive symptoms. Emotion awareness has a partial, mediatory role in these complex relationships. In particular, the bodily awareness of emotions and the ability to differentiate emotions seem to work together to open indirect pathways from attachment to depressive symptoms. More research is needed to clarify the mechanisms involved.

## Figures and Tables

**Table 1:** Descriptive statistics and Pearson’s correlations between age, attachment patterns, emotion awareness dimensions, and internalizing problems (*n* = 223).

	*M*	*SD*	1	2	3	4	5	6	7	8	9	10	11
1—Age	13.51	1.13											
Attachment													
2—Secure	33.56	4.25	−0.13										
3—Anxious/Ambivalent	21.00	7.92	0.02	0.02									
4—Avoidant	24.42	10.10	0.04	−0.13	0.30 ***								
Emotion Awareness													
5—Differentiation	2.15	0.44	0.04	0.02	−0.39 ***	−0.19 **							
6—Verbal Sharing	2.00	0.53	−00.08	0.25 ***	−0.27 ***	−0.35 ***	0.32 ***						
7—Not Hiding	1.95	0.45	−0.05	0.25 ***	−0.06	−0.39 ***	0.16 *	0.43 ***					
8—Bodily Unawareness	1.88	0.54	−0.03	−0.09	−0.35 ***	−0.08	0.35 ***	0.11	−0.03				
9—Other’s Emotions	2.70	0.32	0.01	0.42 **	0.05	−0.07	−0.05	0.09	0.13	−0.11			
10—Analyze Emotions	2.44	0.41	0.03	0.26 **	0.09	0.01	−0.13	0.07	0.21 **	−0.27 ***	0.40 ***		
Internalizing problems													
11—Depression	100.21	90.65	0.04	−0.27 **	0.44 ***	0.27 ***	−0.46 ***	−0.26 ***	−0.25 ***	−0.31 ***	−0.17 **	−0.02	
12—Anxiety	70.96	80.60	−0.06	−0.22 **	0.37 ***	0.31 ***	−0.38 ***	−0.21 **	−0.19 **	−0.36 ***	−0.17 *	−0.02	0.71 ***

* *p* < 0.05; ** *p* < 0.01; *** *p* < 0.0010.

**Table 2:** Descriptive statistics and gender differences for age, attachment patterns, emotion awareness dimensions, and internalizing problems (*n* = 223).

	Female		Male			
	*M*	*SD*	*M*	*SD*	*t* (221)	*d*
1—Age	13.39	1.06	13.66	1.20	−1.73	0.238
Attachment						
2—Secure	33.84	4.03	33.19	4.51	1.13	0.152
3—Anxious/Ambivalent	22.08	8.29	19.57	7.19	2.37 *	0.323
4—Avoidant	24.31	4.67	24.58	5.63	−0.39	0.052
Emotion Awareness						
5—Differentiation	2.09	0.43	2.23	0.44	−2.40 *	0.322
6—Verbal Sharing	1.99	0.51	2.01	0.55	−0.34	0.038
7—Not Hiding	1.96	0.47	1.95	0.43	0.13	0.022
8—Bodily Unawareness	1.80	0.51	1.98	0.57	−2.36 *	0.332
9—Other’s Emotions	2.78	0.27	2.60	0.36	3.98 ***	0.566
10—Analyze Emotions	2.48	0.38	2.38	0.44	1.69	0.243
Internalizing problems						
11—Depression	10.57	9.58	9.72	9.77	0.64	0.088
12—Anxiety	7.57	8.49	8.47	8.76	−0.78	0.104

* *p* < 0.05; *** *p* < 0.001.

**Table 3:** Serial mediation analyses: Total, direct, and indirect effects of each pattern of attachment on depression, and intermediate paths.

Attachment/Path	Depression		
	Effect	*SE*	95% *CI*
Secure			
a_1_	−0.019	0.008	[−0.035, −0.004]
a_2_	−0.001	0.007	[−0.014, 0.013]
Total effect (c)	−0.323	0.117	[−0.554, −0.092]
Direct effect (c’)	−0.334	0.112	[−0.564, −0.097]
Indirect effects			
Total	0.011	0.033	[−0.059, 0.074]
a_1_b_1_	−0.002	0.019	[−0.044, 0.034]
a_2_b_2_	0.002	0.028	[−0.059, 0.056]
a_1_d_21_b_2_	0.011	0.008	[0.001, 0.030]
Anxious/ambivalent			
a_1_	−0.014	0.004	[−0.023, −0.006]
a_2_	−0.012	0.004	[−0.020, −0.004]
Total effect (c)	0.249	0.069	[0.112, 0.386]
Direct effect (c’)	0.196	0.068	[0.066, 0.334]
Indirect effects			
Total	0.053	0.024	[0.010, 0.103]
a_1_b_1_	−0.002	0.013	[−0.031, 0.024]
a_2_b_2_	0.047	0.021	[0.011, 0.092]
a_1_d_21_b_2_	0.008	0.005	[0.001, 0.021]
Avoidant			
a_1_	0.008	0.007	[−0.006, 0.022]
a_2_	−0.004	0.006	[−0.016, 0.007]
Total effect (c)	0.011	0.090	[−0.166, 0.188]
Direct effect (c’)	−0.003	0.087	[−0.176, 0.166]
Indirect effects			
Total	0.014	0.028	[−0.032, 0.080]
a_1_b_1_	0.001	0.010	[−0.018, 0.023]
a_2_b_2_	0.017	0.026	[−0.024, 0.081]
a_1_d_21_b_2_	−0.005	0.005	[−0.017, 0.003]
Common paths			
b_1_	0.116	0.901	[−1.611, 1.919]
b_2_	−3.962	1.120	[−6.161, −1.771]
d_21_	0.143	0.052	[0.041, 0.246]

*Note*. Unstandardized coefficients/estimates. Standard error and 95% confidence intervals provided by bootstrap, except for attachment total effects. An effect is significant when the confidence interval does not include zero.

## Data Availability

Not applicable.
